# Serum Stem Cell Growth Factor Beta for the Prediction of Therapy Response in Hepatocellular Carcinoma

**DOI:** 10.1155/2018/6435482

**Published:** 2018-08-30

**Authors:** Caecilia H. C. Sukowati, Riccardo Patti, Devis Pascut, Rusdina B. Ladju, Paola Tarchi, Nunzia Zanotta, Manola Comar, Claudio Tiribelli, Lory S. Crocè

**Affiliations:** ^1^Fondazione Italiana Fegato, AREA Science Park Basovizza, Trieste, Italy; ^2^Department of Medicine, Surgery, and Health Sciences, University of Trieste, Trieste, Italy; ^3^Faculty of Medicine, University of Hasanuddin, Makassar, Indonesia; ^4^Teaching Hospital, ASUITS, Trieste, Italy; ^5^Institute for Maternal and Child Health, IRCCS Burlo Garofolo, Trieste, Italy

## Abstract

**Introduction:**

Chronic inflammatory response is one of major contributors in the development of hepatocellular carcinoma (HCC). Inflammatory molecules, such as cytokines and growth factors in the circulation, can be useful in the diagnosis and prognosis of the patients. The stem cell growth factor beta (SCGF*β*), a newly found protein, is a secreted sulfated glycoprotein and it functions as a growth factor for primitive hematopoietic progenitor cells. The level of SCGF*β* had been reported to be elevated in several cancer types. However, there is very few or even no information on this protein in the study of HCC, even more in clinical studies.

**Methods:**

A multiplex immunoassay panel of 48 cytokines and growth factors were utilized to screen 68 sera from 29 HCC patients at pretreatment (T0), 1 month (T1), and 6 months (T6) after treatment by either radiofrequency ablation (RF) or transarterial chemoembolization (TACE). Treatment response was evaluated according to mRECIST criteria.

**Results:**

Immunoassay screening showed that the levels of IL-17, CTACK, TNF*α*, IL-2R*α*, IL-8, and SCGF*β* were different in Complete Responders (CR) and Nonresponders (NR) groups. At T0 and T1, the SCGF*β* level was significantly the highest in NR (23.8 and 40.7 ng/mL, respectively), followed by early recurrence (25.4 and 25.0 ng/mL), and CR (6.7 and 5.3 ng/mL), independently from HCV, stages, and treatment type. Low basal SCGF*β* level was associated with longer disease-free survival compared to high SCGF*β*.

**Conclusion:**

In this study, for the first time, we demonstrate that the high level of serum SCGF*β* at pre- and posttreatment is associated with HCC nonresponsiveness.

## 1. Introduction

Liver cancer is one of the most common cancers, being the second cause of cancer related death worldwide. The prognosis of this cancer is poor and the geographical patterns in incidence and mortality are similar [1]. Hepatocellular carcinoma (HCC) accounts for about 90% of liver cancer cases, with cirrhosis as the strongest underlying condition [2]. International guideline recommends image-guided radiofrequency (RF) ablation as the treatment of choice for HCC patients with early-stage HCC when liver transplantation or hepatectomy is precluded. For patients in intermediate stage, palliative treatment by using transarterial chemoembolization (TACE) is recommended [3]. However, in spite of growing technology advances in the cancer diagnosis and treatment techniques, the long-term survival of the patients is still inadequate because of the high rates of cancer recurrence [4].

It is known that chronic inflammatory response is a main risk factor for HCC progression [5], deriving both from viral and metabolic etiological factors of the disease. Inflammatory molecules, such as cytokines and trophic growth factors, can be useful in the diagnosis and prognosis of the patients. Different serum biomarkers can be used to predict the prognosis of patients with unresectable HCC [6–8], in patients who received RF and TACE therapy [9–11], and in patients with advanced stages treated with Sorafenib [12, 13]. However, only few studies have shown the use of serum biomarkers to predict an early recurrence of the cancer and the patients' response. Furthermore, due to the diversity of stimulated cytokines, most of the reported studies used a rather homogeneous group receiving a similar therapy.

The stem cell growth factor (SCGF) is a protein encoded by gene CLEC11A, a member of the C-type lectin superfamily. SCGF is a secreted sulfated glycoprotein and functions as a growth factor for primitive hematopoietic progenitor cells. SCGF is expressed in two distinct forms; SCGF*α* is the full size form (35 kDa), while SCGF*β* is the shorter form (27 kDa) characterized by a deletion within a conserved carbohydrate recognition domain. In primary breast cancer, the level of SCGF*β* was elevated in the circulating tumor cells (CTC) in mononuclear cells of peripheral blood [14]. In lung cancer, drug-resistant cancer stem cells- (CSC-) derived tumors with marker CD133+ contained two- to threefold higher levels of human angiogenic and growth factors, including the SCGF*β* [15]. However, there is very few or even no information on this protein on the study of HCC, even more in HCC patients treated with RF and TACE.

This study reports on the screening of 48 cytokines and trophic factors involved in cancer development in predicting the response of the patients in both early and intermediate stages of HCC receiving RF and TACE. We show that SCGF*β* level can be used to predict HCC recurrence after either RF or TACE.

## 2. Materials and Methods

### 2.1. Patient Selection and Classification

Patients referring to the Liver Center eligible for the assigned treatment protocols of TACE and RF were recruited in the study. The diagnosis of HCC was formulated based on standard radiologic findings by computerized tomography and magnetic resonance imaging scan when the typical imaging features were present [3].

Based on the HCC recurrence, patients were classified into two main groups: patients without any HCC recurrence at 6 months after treatment (T6) were defined as Complete Responders (CR, n=13), while Nonresponders (NR, n=16) were the patients with HCC recurrence within 6 months. The NR group was then subclassified into a group with HCC appearance/persistency in the first month (unsuccessful therapy; NR_T1_, n=5) and another group with HCC recurrence between 1 and 6 months (NR_T6_, N=11). Patients were further defined according to gender, age, previous treatment (TACE, RF, and liver resection); etiology (hepatitis C virus (HCV), hepatitis B virus (HBV), alcohol, and metabolic); laboratory finding (levels of alpha fetoprotein (AFP), alanine aminotransferase (ALT), and aspartate aminotransferase (AST)); and disease score (Barcelona Clinic Liver Cancer (BCLC) and Child Pugh Turcotte (CPT)).

Written informed consent was obtained from patient or by a legal representative and patient anonymity has been preserved. Investigation was conducted according to the principles expressed in the Declaration of Helsinki. The regional ethical committee (Comitato Etico Regionale Unico of the Friuli Venezia Giulia, Prot. No. 18854) approved the study.

### 2.2. Quantification of Serum Cytokines

Sera were collected on the day just before treatment (T0), at 1 month (T1) and 6 months (T6) after treatment. Samples were stored at -80°C until quantification. In brief, 50 *μ*L of serum and standard was added in a 96-multiwell plate containing analyte beads (Bio-Plex, BIO-RAD Laboratories, Milan, Italy) based on a magnetic bead multiplex immunoassays. After incubation for 30 minutes at room temperature and washing, the antibody-biotin reporter was added and incubated for 10 minutes with streptavidin-phycoerythrin. The concentrations of the cytokines were determined using the Bio-Plex array reader (Luminex, Austin, TX). The Bio-Plex Manager software optimized the standard curves automatically and returned the data as Median Fluorescence Intensity (MFI) and concentration (pg/ml). This assay has a reported limit of detection of 1–20 pg/ml, depending on the cytokine target. Each sample was run in a blind fashion, and the value of all individual data was collected for the analysis.

The screening was carried out for complete panel of 48 cytokines, chemokines, and trophic factors (21-plex and 27-plex BioRad). Cytokines analyzed were IL-1*α*, IL-2Ra, IL-3, IL-12p40, IL-16, IL-18, IFN- *α*2, LIF, MIF, SCF, TNF-*β*, TRAIL/TNFSF10, IL-1*β*, IL-1Ra, IL-2, IL-4, IL-5, IL-6, IL-9, IL-10, IL-12p70, IL-13, IL-15, IL-17, IFN-*γ*, and TNF-*α*. Chemokines analyzed were CTACK/CCL27, GRO*α*/CXCL1, MCP-3/CCL7, MIG/CXCL9, SDF-1*α*/CXCL12, IL-8/CXCL8, Eotaxin/CCL11, MCP-1/CCL2, MIP-1*α*/CCL3, IP-10/CXCL10, MIP-1*β*/CCL4, and RANTES/CCL5. Trophic factors analyzed were HGF, M-CSF, *β*-NGF, SCGF-*β*, IL-7, FGF basic, G-CSF, PDGF-b, VEGF, and GM-CSF. All of mentioned factors had been reported to have important roles in cancer development, progression, or metastasis.

### 2.3. Survival Analysis

Recurrence and survival were defined as the time elapsed between treatment of HCC and the first detection of HCC reappearance and death, respectively. For analysis, recurrence and survival of patients with low and high level of serum SCGF*β* were compared using Kaplan-Meier curves and the log-rank test. Cutoff value was defined by a Receiver Operating Characteristic (ROC) curve plotting the true positive rate (sensitivity) against false positive rate (100 - specificity).

### 2.4. Statistical Analysis

The values of serum cytokines were mentioned as median (min-max). Statistical comparison between groups was performed using software InStat Version 3.05 (GraphPad Software, Inc., La Jolla, CA, USA) and IBM SPSS statistics program version 24 (IBM Corporation, New York, USA). Statistical significance was set to p<0.05.

## 3. Results

### 3.1. Basal Cytokine Concentration

The complete clinical and pathological characteristics of the patients enrolled in the study are listed in Table 1. A total of 68 sera samples from 29 HCC patients were recruited with prevalence of gender M22/F7 with a mean age of 70 years (min 53, max 83), 9 with HCV infection, and 20 with alcohol or metabolic etiologies. RF ablation and TACE were performed in 15 patients and 14 patients, respectively. HCC score was mainly CPT score A (29 patients) and BCLC A and B (17 and 10 patients, respectively). The mean of basal AFP and ALT level was 17.8 ± 24.1 ng/mL and 48.0 ± 45.5 U/L, respectively.

By using a screening of 48 cytokines and growth factors in a Luminex platform, our data on the differential cytokines with the basal clinical and pathological parameters is shown in Table 2. We observed that, in HCV-related HCC, the levels of IP-10, IL-2R*α*, and MIG were significantly increased, with the IP-10 level showing the highest difference for around 3-fold (median value 950 [398 – 2586] and 2606 [1478 – 7454], for HCV- and HCV+ HCC, respectively) (p<0.001). As for HCC staging, the level of serum IL-8 was found to be around two-times higher in BCLC B (25.4 [10.3 – 53.9]) compared to BCLC A group (11.9 [2.3 – 23.9]) (p<0.05)]. As for the treatment group, we found that significant difference between group TACE and RF was noticed only for IL-15 and SDF-1a (p<0.01).

### 3.2. Serum SCGF*β* Level to Predict HCC Recurrence

**Figure 1:**
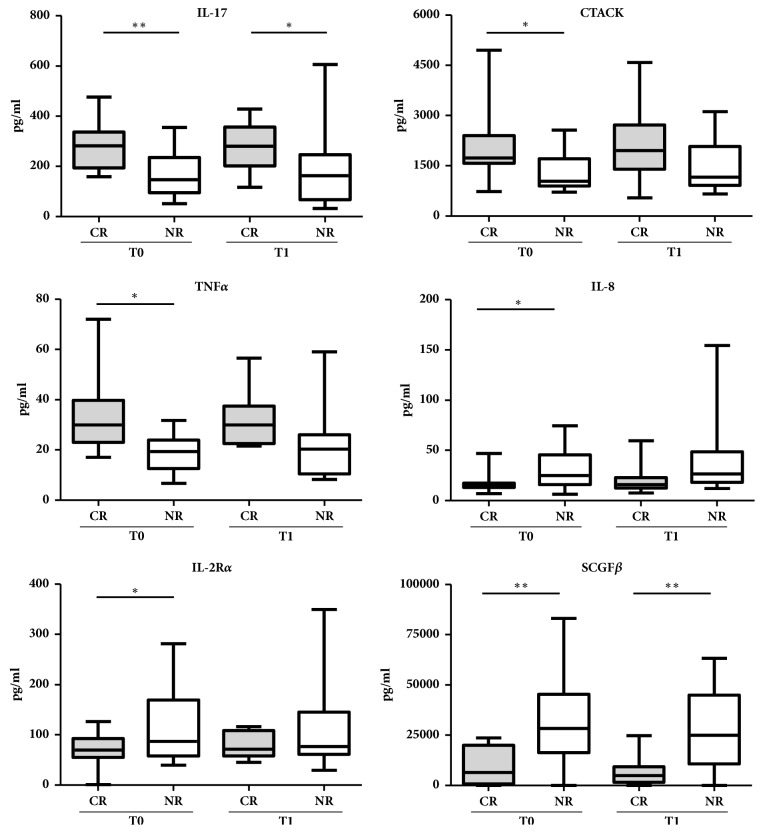
**Differential levels of serum cytokines between Complete Responders (CR) and Nonresponders (NR) patients**. Serum was drawn at before treatment T0 and at 1 month after treatment T1. Cytokines concentration was expressed as median (min-max); statistical significance was calculated by using Student's t-test. *∗* p<0.05, *∗∗* p<0.01.

To study the correlation between the level of cytokines/growth factors and HCC recurrence, we compared the CR and NR groups at pretreatment (T0) and at 1 month after treatment (T1). We found various cytokines and growth factors to be differently expressed in CR compared to NR patients group. Several factors such as IL-17, CTACK, and TNF*α* were increased in CR patients, while IL-2R*α*, IL-8, and SCGF*β* were higher in NR patients. Among these differentiated factors, we found that the SCGF*β* could distinguish the CR and NR groups with a high significant value (p<0.01) (Figure 1).

**Figure 2:**
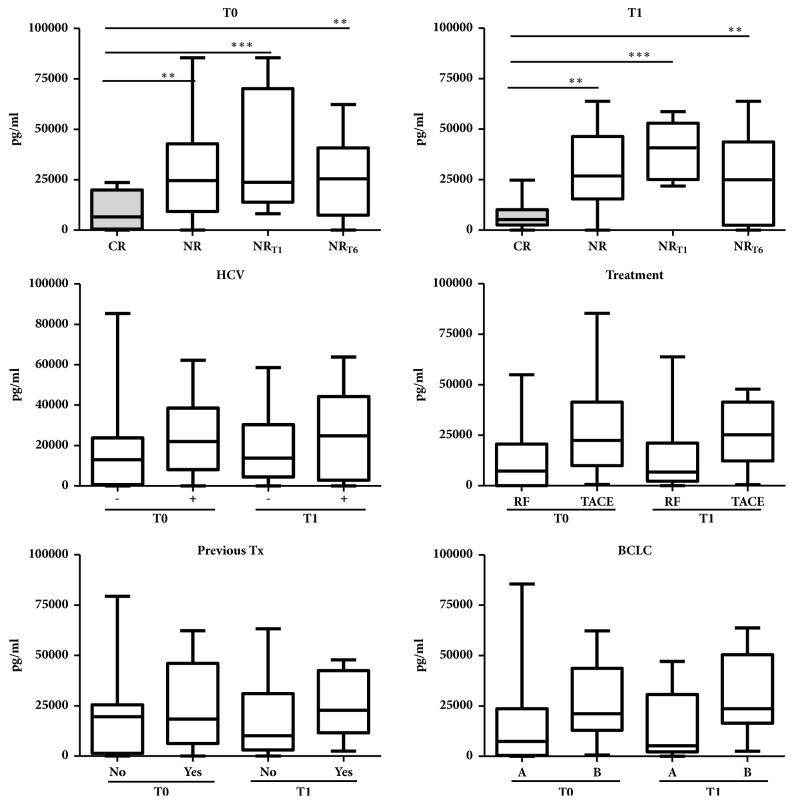
**Correlation of SCGF** **β** **level with clinical-pathological data.** SCGF*β* concentration was expressed as median (min-max); statistical significance was calculated by using Student's t-test. HCV: hepatitis C virus, TACE: transarterial chemoembolization, RF: radiofrequency ablation, and BCLC: Barcelona Clinic Liver Cancer. *∗∗* p<0.01, *∗∗∗* p<0.001.

We performed further analysis by subclassifying the NR group into two groups of patients based on early or later HCC recurrence, as mentioned in Materials and Methods. We found that both at T0 and T1, the level of SCGF*β* was found to be the highest in the group of patients who had unsuccessful treatment (NR_T1_), followed by the group of patients with HCC recurrence between 1 and 6 months (NR_T6_), and the lowest in the group of patients without HCC recurrence at 6 months after treatment (CR). At T0 and T1, the median values obtained were 23.8 and 40.7 ng/ml for NR_T1_, 25.4 and 25.0 ng/ml for NR_T6_, and 6.7 and 5.3 ng/ml for CR group. Collectively these results indicate that the level of SCGF*β* could predict whether a patient would be disease-free between 1 and 6 months after initial treatment (Figure 2).

### 3.3. Serum SCGF*β* Level as a Prognostic Value

At both T0 and T1, the predicting value of SCGF*β* was found to be independent from HCV infection and the history of previous treatments received by the patients. Even though the level of SCGF*β* was slightly higher in the group of patients who would receive TACE compared to ones with RF at T0, and in BCLC B compared to BCLC A, it did not reach any significant values (Figure 2). Our results had shown that the level of SCGF*β* was not correlated with Milan and Up-to-7 criteria. Further, the level of SCGF*β* was not correlated with total volume of the tumor. However, it might indicate that the level of SCGF*β* increases in accordance with the severity of the disease.

**Figure 3:**
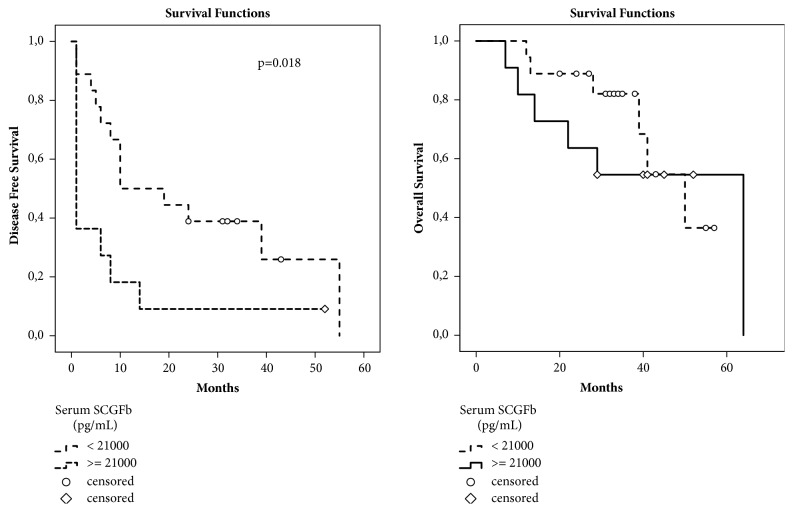
**Significance of basal SCGF** **β** **with prognosis**. Kaplan-Meier analysis of HCC recurrence and overall survival. At T0, serum SCGF*β* concentration higher than 21000 pg/mL is associated with a shorter recurrence time compared to concentration less than 21000 pg/mL.

Kaplan-Meier analysis was performed to associate the level of serum SCGF*β* with HCC recurrence and overall survival (OS) (Figure 3). By using a ROC curve plotting sensitivity/specificity, a cutoff value of 21000 pg/mL was defined with 79% sensitivity and 80% specificity (data not shown). From the analysis of HCC recurrence, we found that patients with serum SCGF*β* higher than 21000 pg/ml had shorter time to tumor recurrence compared to patients with lower serum SCGF*β* (mean value 7.9 ± 4.4 month versus 24.8 ± 5.3 month, respectively, p=0.018).

For OS analysis, the means of OS on both groups were comparable for around 40 months (42.4 ± 8.0 versus 44.1 ± 3.9 month, respectively, p=ns). Nevertheless, the difference on the OS probability was clearly noticed between 10 and 40 months. The analysis on RF patients alone showed a similar trend (data not shown). This data indicated that SCGF*β* has a positive prognostic value.

## 4. Discussions

We profiled 48 cytokines in 68 HCC sera samples in order to select predictive biomarkers for therapy response and disease recurrence. To our knowledge, no similar studies have been previously reported.

As expected, in untreated patients, various cytokines were expressed differently based on HCV positivity, previous treatments, and the stage of the disease. In HCV-related HCC, we observed significant differences on IP-10, IL-2Ra, and MIG, with the highest for the IP-10 (10kDa interferon gamma-induced protein), a proinflammatory protein. These findings are in line with previous studies showing high concentration of IP-10 in HCV-related liver diseases such as acute C infection, chronic hepatitis, fibrosis, and HCC [16, 17]. IP-10 has been used to predict the response of antiviral therapy for HCV infection [18, 19].

We observed that the SCGF*β* is one of the most significant biomarkers in predicting early HCC recurrence after RF and TACE. The level of SCGF*β* was found to be the highest in the group of patients who had unsuccessful treatment, followed by the group of patients with early HCC recurrence (between 1 and 6 months), while the lowest is in the group without HCC recurrence at 6 months after treatment. Interestingly, this predicting factor is independent from HCV infection, BCLC stages, and previous treatment history. More importantly, we found that the basal level T0 of serum SCGF*β* has a positive prognostic value with HCC recurrence. Patients with high serum SCGF*β* (>21000 pg/ml) has a higher probability for an early HCC recurrence compared to those with lower level of SCGF*β*.

Even though we did not see a statistical difference on the OS, a separated analysis on RF and TACE group alone showed similar trend. The group of SCGF*β* level higher than 21000 pg/ml showed worse OS than the group with lower level. Interestingly, since RF is a curative treatment, this result indicates that, in the presence of high level of SCGF*β*, a curative therapy was still ineffective, thus supporting the importance of SCGF*β* as a prognostic biomarker. We also noticed that the curves began to separate only after 10 months posttreatment. This pattern was caused by the maintenance effect of treatment or reprocessing.

Until now, limited information is available regarding this protein. The SCGF is a protein encoded by gene CLEC11A, a member of the C-type lectin superfamily. SCGF was selectively produced by osseous and hematopoietic stromal cells and can mediate their proliferative activity on primitive hematopoietic progenitor cells [20, 21]. It was also demonstrated that beside the property of SCGF as one of the stem cells markers, leukemic cells also required self-secreted SCGF for their proliferation, indicating an autocrine or paracrine SCGF regulation of hematopoietic stem/progenitor cells [22]. A recently published CoMMpass study demonstrated that, by using an integrative network biology analysis, CLEC11A was found to be a novel regulator and a candidate of therapeutic target in in multiple myeloma [23].

In a very recent HCC study, a series of cytokines, including the SCGF*β*, IL-6, CCL2/MCP-1, CXCL1/GRO*α*, CXCL8/IL-8, HGF, and VEGF, were secreted from peritumor tissue-derived fibroblasts and was shown to promote development of HCC by recruiting CSC and maintaining their stemness characteristic [24]. In lymphoblastic leukemia, by using microarray analysis of 35 matched diagnosis/relapse pairs, as well as 60 uniformly treated children at relapse, the SCGF was found to be a gene significantly overexpressed at relapse [25]. In chemotherapy-naive testicular germ-cell tumor patients, elevated levels of SCGF*β* were associated with worse overall survival [26]. SCGF*β* was also found to be increased among different immune mediators in the sera of the asbestos-exposed workers compared to controls [27]. In this report, consistently with other published studies, we show that the levels of SCGF*β* were increased in the groups with early HCC recurrence.

## 5. Conclusion

To summarize, we have demonstrated that the high level of serum SCGF*β* pre- and post-treatment is associated with HCC nonresponsiveness and early recurrence after either RF or TACE treatment. These data indicate the significance of the growth factors of stem/progenitor cells in HCC.

## Figures and Tables

**Table 1:** Clinical-pathological characteristics of the patients enrolled in the study.

	**RF**	**TACE**	**TOTAL**	**p**
Patients characteristics				

Male / Female	11/4	11/3	22/7	ns
Age	72 ± 5	68 ± 10	70 ± 8	0.010
Etiologies	15	14	29	ns
Alcohol/metabolic	9	7	16	
Alcohol	1	3	4	
HCV	5	4	9	

Clinical characteristics				

CTP A/B	10/0	15/4	25/4	0.042
BCLC 0/A/B	2/10/3	2/5/7	4/15/10	ns
Milan criteria (in/out)	13/1	8/3	21/4 (unknown 4)	ns
Up-to-7 criteria (in/out)	14/0	9/2	23/2 (unknown 4)	ns
Largest nodule diameter (mm)	29 ± 10	27 ± 7	28 ± 9	ns
Total tumor volume (cm^3^)	18 ± 24	22 ± 25	20 ± 24	ns

Laboratory finding				

Creatinine (mg/dl)	0.9 ± 0.3	0.8 ± 0.2	0.9 ± 0.2	ns
Sodium (mmol/l)	136.0 ± 2.2	138.4 ± 2.6	138.0 ± 2.4	ns
Total bilirubin (mg/dl)	0.94 ± 0.33	1.33 ± 0.93	1.12 ± 0.68	0.033
Total protein (g/dl)	7.2 ± 0.5	7.4 ± 0.7	7.2 ± 0.6	ns
Albumin (g/dl)	3.8 ± 0.4	3.6 ± 0.3	3.7 ± 0.3	ns
AST (IU/l)	77.7 ± 79.7	46.7 ± 24.3	63.9 ± 62.5	0.001
ALT (IU/l)	80.3 ± 96.7	37.4 ± 19.4	61.2 ± 75.3	0.001
ALP (IU/l)	101.3 ± 44.1	101.8 ± 40.7	101.5 ± 41.8	ns
GGT (IU/l)	112.4 ± 109	135.3 ± 146.5	123.4 ± 126.7	ns
WBC (10^3^/*μ*l)	5.1 ± 2.1	4.8 ± 2.1	4.97 ± 2.07	ns
Hemoglobin (g/dl)	13.5 ± 2.1	13.7 ± 1.5	13.6 ± 1.8	ns
Hematocrit (%)	40.4 ± 5.7	40.6 ± 4.3	40.5 ± 4.9	ns
Platelets (10^3^/*μ*l)	135.7 ± 80	102.6 ± 55.3	119.7 ± 70.2	ns
AFP (ng/ml)	20 ± 30.7	18.5 ± 23.3	19.3 ± 26.8	ns
INR	1.12 ± 0.1	1.18 ± 0.1	1.14 ± 0.12	ns

HCV: hepatitis C virus, CTP: Child-Turcotte-Pugh, BCLC: Barcelona Clinic Liver Cancer, AST: aspartate aminotransferase, ALT: alanine aminotransferase, ALP: alkaline phosphatase, GGT: gamma glutamyl transferase, WBC: white blood cells, AFP: alpha fetoprotein, INR: international normalized ratio, and ns: not significant

**Table 2:** Basal levels of circulating cytokines in HCC patients.

	**Cytokine level (pg/ml) [median (min-max)]**		
Viral infection	HCV+ (n=10)	HCV- (n=19)	p
IP-10	2606 (1478 – 7454)	950 (398 – 2568)	<0.001
IL-2R*α*	117 (58 – 284)	65 (1 – 182)	<0.05
MIG	1029 (468 – 1550)	551 (70 – 1740)	<0.01

Treatment choice	RF (n=15)	TACE (n=14)	p

IL-15	21.5 (7.2 – 44.6)	11.6 (0.2 – 27.8)	<0.01
SDF-1a	344 (157 – 552)	511 (218 – 982)	<0.01

BCLC stage	A (n=17)	B (n=10)	p

IL-8	15 (6 – 47)	25 (10 – 54)	<0.05
HGF	409 (283 – 1111)	590 (363 – 1649)	<0.05

Quantification of the serum cytokines was determined by using Bio-Plex kit (Bio-Rad) in Bio-Plex array reader (Luminex, Austin, TX). Cytokines levels were mentioned as median (min-max); statistical significance was calculated by using Student's t-test. HCV: hepatitis C virus, TACE: transarterial chemoembolization, RF: radiofrequency ablation, and BCLC: Barcelona Clinic Liver Cancer.

## Data Availability

The data used to support the findings of this study are available from the corresponding author upon reasonable request.
